# A new F-18 labeled PET tracer for fatty acid imaging

**DOI:** 10.1007/s12350-014-0012-4

**Published:** 2014-10-25

**Authors:** Fabian Demeure, Manuel D. Cerqueira, Michel Hesse, David Vancraeynest, Véronique Roelants

**Affiliations:** 1 Pôle de Recherche Cardiovasculaire, Institut de Recherche Expérimentale et Clinique, Université catholique de Louvain, Brussels, Belgium; 2 Division of Cardiology, Cliniques Universitaires Saint-Luc, Brussels, Belgium; 3 Department of Nuclear Medicine, Imaging Institute, Cleveland Clinic, Cleveland Clinic Lerner College of Medicine of Case Western Reserve University, 9500 Euclid Ave, Jb-3, Cleveland, OH 44195 USA; 4 Nuclear Medicine Department, Cliniques Universitaires Saint-Luc, Avenue Hippocrate, 10, 1200 Brussels, Belgium; 5 Pôle d’Imagerie moléculaire, Radiothérapie et Oncologie, Institut de Recherche Expérimentale et Clinique, Université catholique de Louvain, Brussels, Belgium

In the presence of acute or chronic myocardial ischemia, myocardial energy metabolism is shifted from the use of aerobic fatty acids (FA) substrate to anaerobic glucose utilization resulting in decreased FA uptake in ischemic regions. This phenomenon may persist as long as 24-30 hours following an acute ischemic event, despite restoration of blood flow and resolution of symptoms and is referred as “ischemic memory”.^1^ Ischemic memory imaging can be particularly useful in various clinical settings^2^ as in the case of patients presenting to the emergency department with acute chest pain syndrome.^3^ We present the first human images of CardioPET (trans-9-F-18-fluoro-3,4-methyleneheptadecanoic acid, FCPHA)^4^ a F-18 labeled modified FA currently being evaluated in a phase II multicentre clinical trial in patients with a positive SPECT stress test for ischemia.

**Figure 1:**
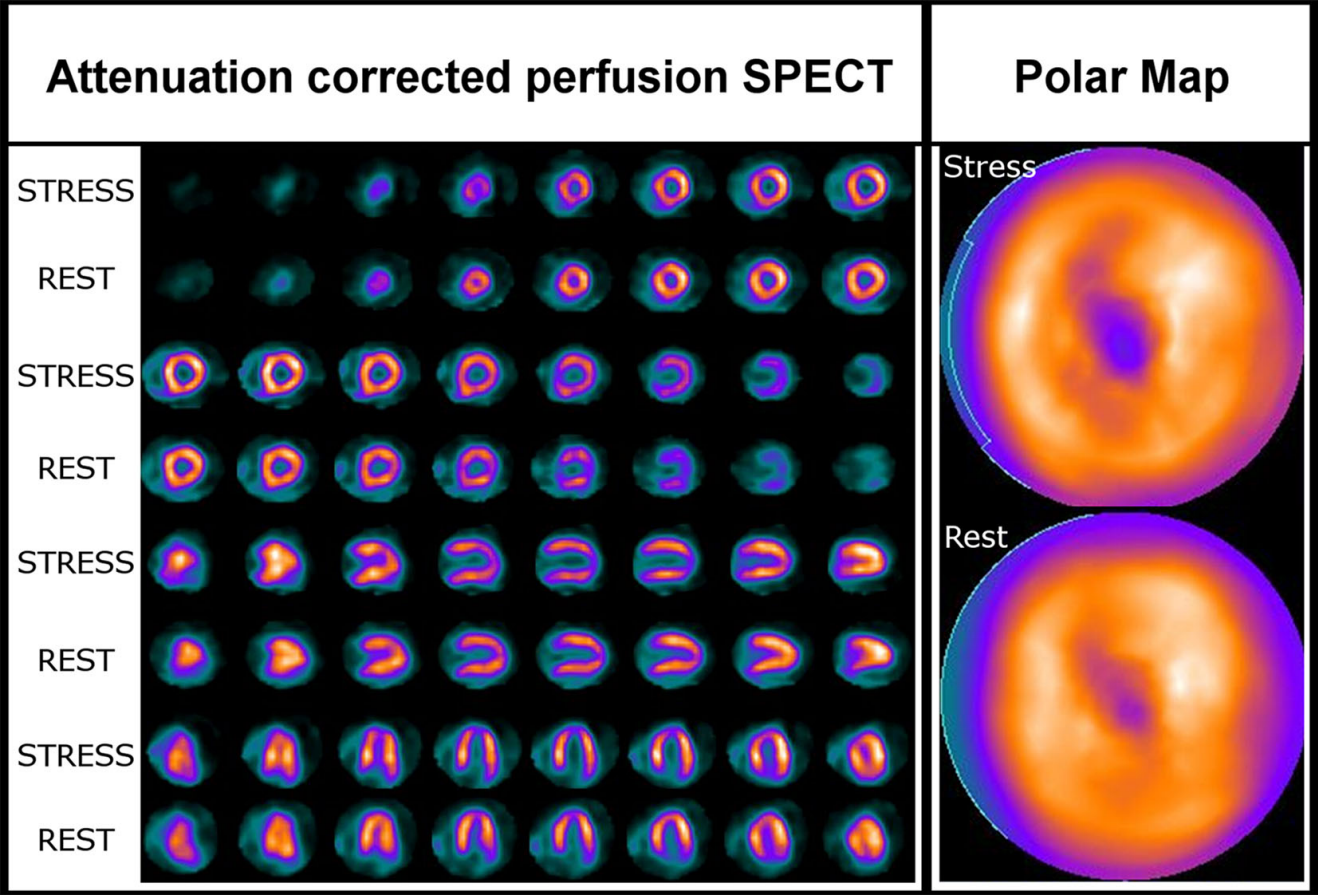
Attenuation corrected stress-rest Tc-99m-Sestamibi perfusion SPECT images. Sestamibi was injected at the peak of the stress test (160 watts, 131 bpm, 86% of maximal predicted heart rate and blood pressure of 210/100 mm Hg). The patient experienced typical angina and ischemic ECG changes in leads V5-V6 during recovery. The SPECT images show a severe apical and moderate antero-apical reversible perfusion defect

**Figure 2:**
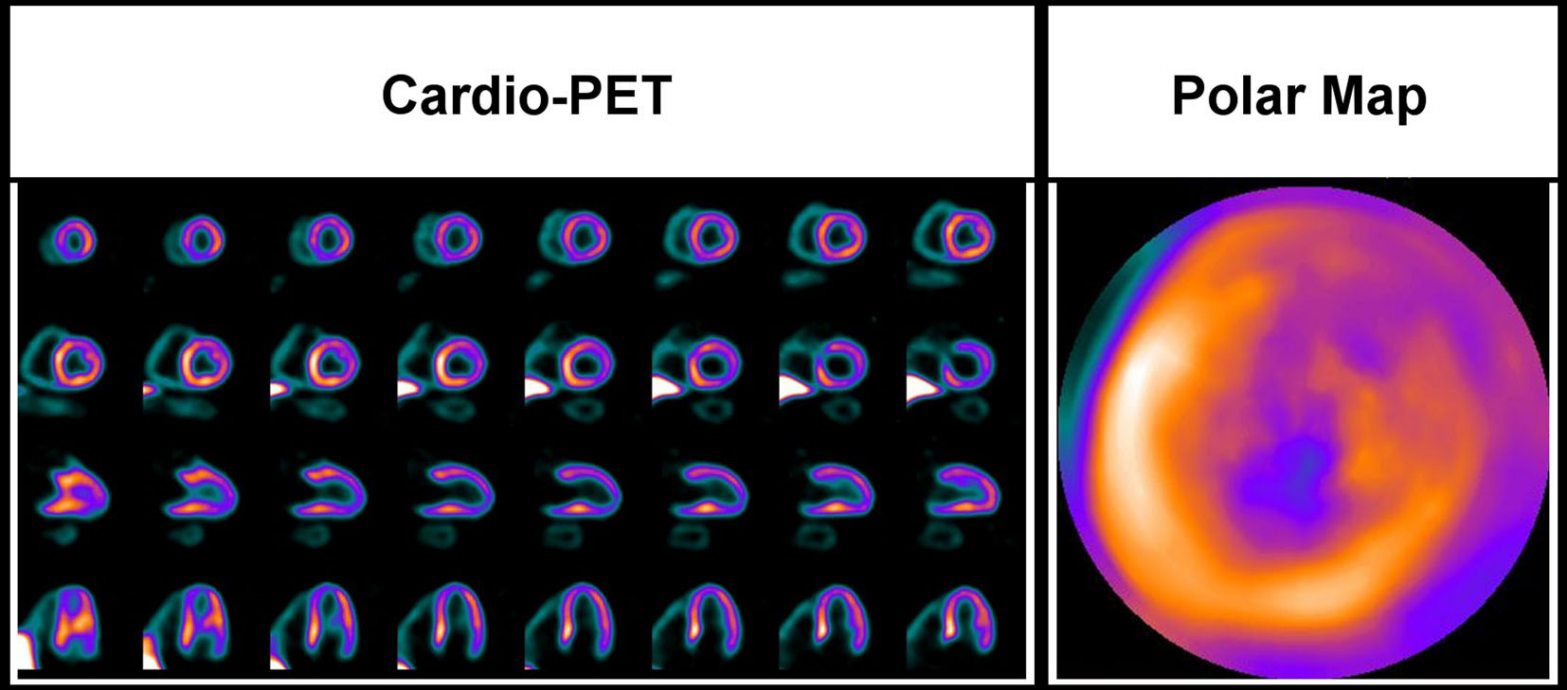
CardioPET images. CardioPET enters the myocardium by the same mechanism as naturally occurring free FAs and then undergoes partial metabolism before being trapped in the myocytes. The tracer (262 MBq) was injected 8 minutes after peak exercise stress and a 60-minute list mode acquisition was performed. Data were reconstructed with dead time, random, scatter, and CT-based attenuation correction. Static 5 minutes images were formatted at 1, 5, 15, 35, 55 minutes post-injection. The 35 minutes post-injection images presented here show a severely decreased tracer uptake in the apical and basal posterior segments of the left ventricle and a moderately decreased tracer uptake in the anterior and anterolateral regions

**Figure 3:**
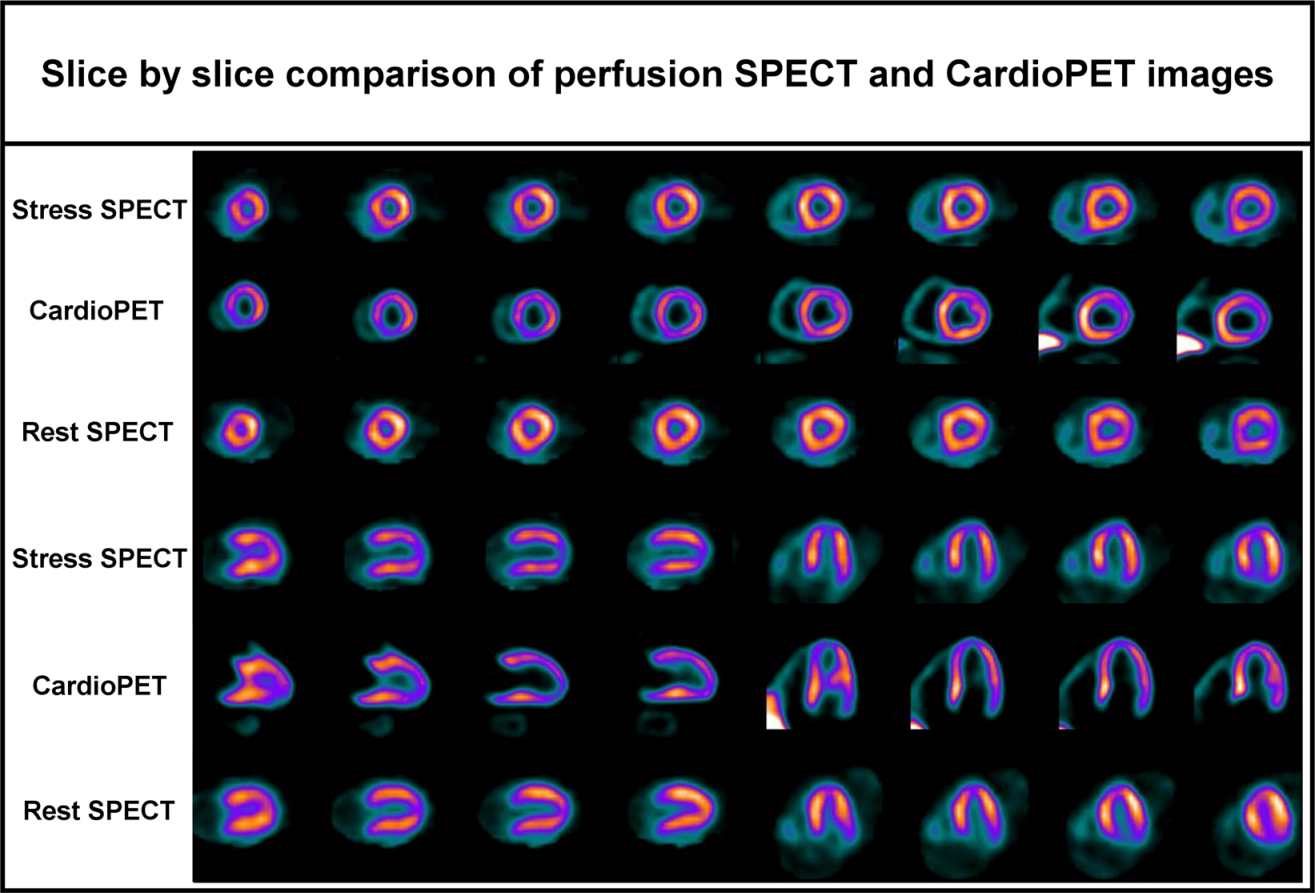
Slice by slice comparison of the stress perfusion SPECT, CardioPET, and rest perfusion SPECT images allowing to appreciate the more extensive defects on the cardioPET scan in relation to the Tc-99m-Sestamibi stress perfusion SPECT scan

**Figure 4:**
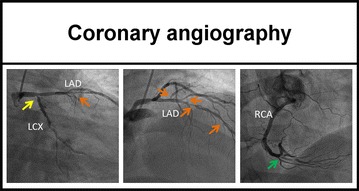
Coronary angiography. Coronary angiography revealed multi-vessel disease with a severe (76%-95%) ostial lesion of the circumflex artery (*yellow arrow*), 2 successive severe (76%-95%) medial lesions of the left anterior descending artery, a severe (76%-95%) ostial first and second diagonal’s lesions (*orange arrows*), and finally a severe (76%-95%) ostial lesion of a small posterior descending artery (*green arrow*). The extent of the coronary disease was better identified by the CardioPET than by the Tc-99m-Sestamibi perfusion SPECT

**Figure 5:**
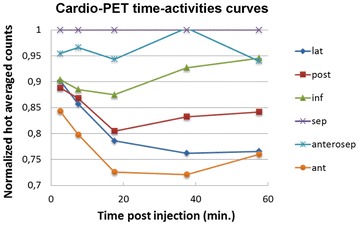
CardioPET time-activities curves. The graph shows the evolution of the activity in the different regions of the left ventricle (lateral, posterior, inferior, septal, anteroseptal, and anterior) as a function of the time after tracer injection. Counts were normalized to the counts of the hottest region, i.e., the septal wall. There is a time-dependent CardioPET uptake which is different in non-ischemic region (anteroseptal, septal, and inferior walls) than in ischemic regions (anterior, lateral, and posterior walls)

A 69-year-old man with typical angina was evaluated using bicycle exercise stress-rest Tc-99m-Sestamibi SPECT (Figure 1). As part of a research protocol, prior to coronary angiography, the patient underwent repeat bicycle stress CardioPET imaging at a similar heart rate. CardioPET was injected 8 minutes after peak exercise stress test. CardioPET images (Figure 2) revealed alterations of fatty acid uptake in regions without abnormalities on Tc-99m-Sestamibi-SPECT (Figure 3). It better identified the extent of significant coronary stenosis than did Tc-99m-Sestamibi SPECT as demonstrated by coronary angiography (Figure 4). In addition, regional time-activity curves obtained from the CardioPET data suggested dynamic time-dependent changes in fatty acid retention in ischemic regions different from the non-ischemic regions that need to be further investigated (Figure 5). These preliminary findings suggest a promising role of this new F-18 labeled tracer of FA uptake for the identification of functionally significant coronary artery disease. FA uptake and retention in various pathophysiological conditions may be better understood using the quantitative capabilities of PET technology.
